# Leisure and Leisure Education as Resources for Rehabilitation Supports for Chronic Condition Self-Management in Rural and Remote Communities

**DOI:** 10.3389/fresc.2022.889209

**Published:** 2022-06-03

**Authors:** Susan Hutchinson, Heidi Lauckner, Christie Stilwell, Brad A. Meisner

**Affiliations:** ^1^School of Health and Human Performance, Dalhousie University, Halifax, NS, Canada; ^2^School of Occupational Therapy, Dalhousie University, Halifax, NS, Canada; ^3^Faculty of Health, Dalhousie University, Halifax, NS, Canada; ^4^School of Kinesiology and Health Science, York University, Toronto, ON, Canada

**Keywords:** chronic condition self-management, leisure, leisure education, rehabilitation services, rural

## Abstract

The potential of leisure (enjoyable free time pursuits) to be a resource for chronic condition self-management (CCSM) is well-established. Because leisure pursuits are often self-determined, they have the potential to allow people to not only address self-management goals (e.g., managing symptoms through movements or stress-reducing activities) but meet important psychosocial needs (e.g., affiliation, sense of mastery) as well as support participation in a range of meaningful life situations. In this “Perspective” piece, we advocate for the ways leisure and leisure education can be resources for rehabilitation professionals to support CCSM, especially in rural and remote communities. In particular, we focus on aspects of the Taxonomy of Everyday Self-Management Strategies [TEDSS (1)] to highlight ways that embedding leisure and leisure education into supports for CCSM can strengthen rehabilitation services offered to rural and remote dwelling adults living with chronic conditions. Recognizing the breadth of leisure-related resources available in rural and remote communities, we recommend the following strategies to incorporate a focus on leisure-based self-management within rehabilitation services: (a) enhance the knowledge and capacity of rehabilitation practitioners to support leisure-based CCSM; (b) focus on coordinated leadership, patient navigation, and building multi-sectoral partnerships to better link individuals living with chronic conditions to community services and supports; and (c) educate individuals with chronic conditions and family/carers to develop knowledge, skills, awareness and confidence to use leisure as a self-management resource.

## Introduction

In this “Perspective” piece we draw on evidence and experience to advocate for the ways leisure should be a more readily used resource for rehabilitation professionals to support chronic condition self-management (CCSM) in rural and remote communities. In particular, we focus on aspects of the Taxonomy of Everyday Self-Management Strategies [TEDSS (1)] to highlight ways that rehabilitation services are well-suited to weave leisure and leisure education into CCSM, which can strengthen supports to rural and remote dwelling adults living with chronic conditions.

## Living With Chronic Conditions in Rural and Remote Contexts

The prevalence of chronic conditions (e.g., diabetes, heart disease, arthritis, mood disorders) is significant. One in every three Canadian adults lives with at least one condition and 12.9% report living with two or more chronic conditions; this prevalence is even higher for people living in rural locations (14.9%) (2). Chronic physical or mental condition self-management involves managing symptoms alongside the management of the psychosocial and practical impacts of the condition on everyday life [e.g., changes in valued activities, roles and relationships (3)]. Chronic condition self-management is often challenging (4), with additional challenges present for those living in rural and remote communities (5). For example, rural-dwelling individuals living with chronic conditions are at greater risk of worsening symptoms and poorer health (e.g., multimorbidities) due to experiencing greater health disparities (e.g., higher rates of poverty, lower levels of education and health literacy) than urban-dwelling individuals (6, 7). One of the greatest disparities is access to health care (5, 8, 9). Sharp et al. (10) identified distance as a key barrier in remote areas, which is further complicated by limited health resources such as healthcare facilities and trained practitioners. Further, lack of awareness about resources as well as limited social supports often result in a “crisis” for rural dwelling older adults transitioning from hospital-based care to living at home (8). Working in collaboration across health and community sectors to support rural dwelling adults in self-managing their chronic condition should be a priority for an overburdened healthcare system in rural and remote contexts (9, 11, 12).

## Expanding Everyday Self-Management Strategies

Support for CCSM tends to be offered through healthcare systems (e.g., medical and rehabilitation practitioners) with the intention that people will continue to implement strategies on their own at home, with check-ins and support from health professionals. However, with challenges related to transportation and limited access to healthcare in rural and remote communities, ongoing supports for CCSM may be limited. As a result, a more comprehensive understanding of self-management strategies is warranted.

The Taxonomy of Everyday Self-Management Strategies (TEDSS) (1) is a recently developed self-management tool that focuses on the specific behaviors and tasks required of individuals to effectively self-manage their chronic condition and aims to provide a comprehensive and unified framework for self-management inclusive of all aspects of everyday life. The TEDSS was developed based on the literature and qualitative data gathered from 117 individuals living with neurological conditions in the community but was designed to be used to assess and support CCSM among people living with all types of chronic conditions.

A key strength of the TEDSS framework is that it broadens the types of daily strategies that support self-management. This is particularly relevant to rehabilitation professions that focus on the functional implications and management of chronic conditions during everyday activities. Uniquely, the strategies in the TEDSS go beyond typical disease management strategies to include strategies related to social interactions, strategies that focus on adapting and prioritizing valued activities, internal strategies related to staying positive and controlling stress, and behavioral strategies that include physical and mental exercises (1). This expanded focus includes a wider range of potential daily activities that can be used in self-management, which may afford people living in rural and remote contexts additional options beyond those mainly reliant on health services. Below, we contend that leisure clearly falls within this wider range of daily activities; an increased recognition of leisure as a relatively accessible and often underused resource for everyday CCSM can help address the challenges faced by rural-dwelling adults and their carers living with chronic conditions.

## Leisure as a Resource for Chronic Condition Self-Management

Although the words leisure and recreation are often used interchangeably, leisure is typically understood to be enjoyable and personally meaningful pursuits or experiences in one's free time (13), with recreation often considered to be more formal organized programs or activities (14) (e.g., yoga class). For brevity, we use leisure as an overarching term. The enjoyable and meaningful nature of a leisure experience is subjectively defined and varies between individuals and contexts. Rural contexts for leisure participation can be diverse, often occurring in community settings (e.g., can occur in more informal settings like bingo halls or legions) (10). Regardless of what leisure activities people engage in, where or with whom, there is considerable evidence of health and wellbeing benefits associated with leisure participation (15, 16).

While there are obvious leisure benefits related to physical wellbeing (17, 18) (e.g., of physically active leisure for maintaining strength and mobility), leisure also supports CCSM through its contributions to aspects of psychosocial wellbeing (19). For example, leisure assists not only with coping with and adapting to lifestyle changes, but also with personal growth following the onset of an acquired disability or chronic condition (20, 21). Even brief or more casual forms of leisure can aid in CCSM (22).

In studies with individuals living with chronic conditions they described strategies they employed that enabled them to effectively use leisure as a self-management resource, including: (a) committing to leisure for health purposes; (b) drawing on existing personal attributes (e.g., positive attitude, previous leisure skills, beliefs in the importance of taking an active role in self-managing one's condition, accepting limitations) and social resources (e.g., family, friends, work colleagues) to stay motivated and involved; (c) using cognitive (e.g., self-talk) or behavioral strategies (e.g., using compensatory aids) to continue to participate in valued activities, even if in modified ways; and (d) setting leisure-based goals that met personal needs (both for maintaining and enhancing health and getting more out of life) (23–25). Hutchinson and Nimrod (22) noted that successful leisure participation strengthened people's perceptions of their abilities to successfully self-manage their chronic condition.

While leisure is not explicitly named in the TEDSS (1), features of leisure and the leisure strategies used by people living with chronic conditions described above are evident in the TEDSS framework. Specifically, TEDSS health behavior strategies explicitly name physical and mental activities such as being physically active and engaging in activities such as games. Further, strategies described above are also seen in TEDSS social interactions strategies (e.g., staying in contact with family and friends), as well as TEDSS internal strategies that involve acceptance and staying positive. Finally, TEDSS activities strategies explicitly mention engaging in valued activities which can and should include leisure and strategies to adapt leisure activities. Explicitly drawing attention to leisure as an essential element of CCSM in general, and within the TEDSS specifically, can provided helpful direction to rehabilitation professionals and the people they serve, particularly in rural and remote contexts.

Despite the congruence between leisure and the TEDSS, leisure participation is often among the first activities to be stopped following functional declines due to chronic conditions (26, 27). Many people living with chronic conditions experience barriers to participating in leisure related to their chronic condition, such as pain and limited energy (25, 27). Limited access to transportation and lack of coordination between sectors (9, 18) serve as additional barriers. From the literature, we also know that rehabilitation professions often prioritize safety and mobility over leisure, even when it is within their domain of practice (28, 29). As a result, many people with chronic conditions accessing rehabilitation services do not see how leisure can support their efforts to self-manage their chronic condition.

While relevant to all people living with chronic conditions, this gap is particularly concerning for those in rural and remote areas where opportunities for diverse informal and formal leisure experiences or programs (e.g., seniors center, service or faith-based groups, legions) might be more accessible than health services (10). During quality improvement discussions with practitioners in community health centers in our region, they similarly noted barriers to leisure participation related to patient concerns about their physical abilities to engage in leisure activities, costs associated with some leisure activities, transportation issues, and lack of knowledge about community resources. Thus, a coordinated effort that focuses on individual knowledge and skill building related to leisure-based self-management strategies is warranted, while simultaneously addressing systems barriers to ensure access to such leisure opportunities (30).

## Leisure Education: A Tool to Support Self-Management in Rural and Remote Contexts

Elsewhere we have advocated for why and how leisure services could be integrated within a broad system of supports and services for people living with chronic conditions, and how available community resources can be used to meet patients' needs (30). We framed these arguments in relation to the Expanded Chronic Care Model (ECCM) (31) and recommended ways to improve patient education and access to recreation opportunities through greater collaboration across sectors, systems-level capacity building regarding the integration of leisure into CCSM services, and ensuring equity issues are brought to the forefront. These recommendations are also relevant for people living in rural and remote communities and can be further advanced through the integration of leisure education as an additional approach rehabilitation professionals can use to support CCSM.

Leisure education applies theory and evidence about leisure and leisure behavior to the design of experientially or learning-oriented activities intended to enhance participants' knowledge, skills, awareness, and confidence related to leisure and recreation participation (32). While the ultimate goal of leisure education is to help people do more of what matters to them in ways that contribute to their overall health and wellbeing, a key focus of leisure education could be to help people develop leisure-based CCSM strategies that can be used in everyday life. Leisure education can also help family and carers develop self-care strategies and with learning new or additional ways to support CCSM (33). There are several examples of leisure education designed to enhance health and wellbeing more generally (34, 35), including leisure education added to the widely available Stanford Chronic Disease Self-Management Program (36). For example, Janke et al. (36) incorporated topics related to leisure's benefits, motivations, barriers, resources and, through their educational module, supported individuals to create a leisure-based action plan. To support the effectiveness of such individual-level leisure education strategies, leisure education is also needed within the rehabilitation and healthcare sector to ensure leisure is recognized as a legitimate and valuable aspect of CCSM and that infrastructure and systems are in place to support such leisure opportunities (37).

## Recommendations to Incorporate Leisure Within the TEDSS and CCSM Supports in Rural and Remote Contexts

Although leisure is already implicitly found in some CCSM programs [e.g., through the use of community activities (18) and the fostering of social activities through peer and group sessions (38)], this needs to be made explicit and strengthened. As Warner et al. noted, “self-management programs do not seem to provide strategies to facilitate individuals' abilities to access needed resources and engage in social interactions or daily activities, which are equally important to living well with a chronic condition. Only 16% of CCSM programs offered strategies to improve individuals' engagement in activities they valued (39).”

To incorporate a focus on leisure-based CCSM within rehabilitation services in rural and remote communities, we propose supplementing strategies for self-management emphasized in the ECCM and the TEDSS with leisure education at patient/client and rehabilitation service provider levels. The following three recommendations are what we believe rehabilitation practitioners can do to amplify the potential of leisure to be a resource for self-management in community contexts, regardless of whether or not the work of the team is guided specifically by the TEDSS. These recommendations are informed by research evidence and our own efforts to bridge community resources and health services and to build the capacities of both service providers, including rehabilitation professionals, and individuals living with chronic conditions.

- *Rehabilitation practitioners educate themselves:* Before educating individuals about leisure and leisure participation for CCSM, clients or patients need access to appropriate supports, including informed health and human service providers as well as accessible and supportive leisure environments (30). This means that service providers—including recreation, rehabilitation, and other allied health service providers—must understand leisure and appreciate the ways leisure can serve as a resource for CCSM. The literature reviewed earlier provides evidence to support this recommendation. With this knowledge, they will be able to more effectively “coach” people living with chronic conditions in all aspects of goalsetting, problem-solving and planning (including planning adaptations) and when seeking and accessing community supports. In addition to educating themselves (and gathering information to share with clients/patients) about the benefits of leisure for health and CCSM, rehabilitation practitioners are strongly encouraged to educate themselves about the breadth of community resources that are available to support leisure-based CCSM, and to give thought to how they might support individuals in becoming educated and informed, and in applying this knowledge to action-planning for CCSM.
- *Focus on coordinated leadership, patient navigation, and building multi-sectoral partnerships:* To support leisure-based CCSM it is important that rehabilitation practitioners adopt a coordinated approach to care management, recognize the importance of serving as a bridge or link to supportive community contexts (i.e., patient navigation), and clearly identify a responsibility for and mandate to build partnerships that support leisure-based CCSM. A coordinated leadership approach requires shared responsibility and mutual influence between individual clients/patients, their family/carers and service providers, resulting in coordinated actions that enhance client/patient engagement and outcomes (40). This also ensures that clients/patients' and family/carers' needs are at the center of education and supports. As it relates to supporting leisure-based CCSM, it is important that rehabilitation practitioners assume responsibilities for “resource linking” or patient navigation so that client/patients and their family/carers are actively supported in connecting to and accessing community-based services and supports. Rehabilitation practitioners are ideally positioned to serve as leaders in this care coordination, which can encompass a chronic disease navigator role (41). Elsewhere we have identified key characteristics of and processes to build strong partnerships (10, 36). Here we want to emphasize the need for practical and creative processes to support community-based leisure engagement by people living with chronic conditions in rural and remote contexts, such as: (a) being creative about where leisure can occur in rural and remote settings, which involves broadening the circle of potential partners; (b) having formal and informal referral processes in place to support resource linking; (c) hosting leisure sampling programs in a range of spaces (so people living with chronic conditions can be ‘exposed' to possibilities); and (d) co-facilitating programs in community spaces with different partners (to help people become more familiar and comfortable with accessing these spaces or programs). In practical terms, the first steps to partnership building requires rehabilitation practitioners to become informed about locally available leisure opportunities and build relationships with people and services outside of the health sector. It is also important that rehabilitation managers allow space and time for this relationship building for their rehabilitation staff and to create the structures and supports for a coordinated leadership approach to patient navigation and case management.
- *Provide leisure education to individuals:* Once grounded in the evidence of leisure as a valuable support for CCSM, education about and for leisure can be shared with people living with chronic conditions and their family/carers so they have access to leisure education as well (20, 23, 24, 33). Individual and group education for CCSM is a recommended practice for CCSM support, especially when it emphasizes goal setting, action planning and problem-solving (42). By extension we recommend that leisure education for CCSM focus on assisting people living with chronic conditions and their family/carers to: (a) become more aware of the benefits of leisure for their health and overall wellness (i.e., develop beliefs about its value for self-managing their chronic condition); (b) understand their own motivations for participation; (c) develop skills (both for specific leisure pursuits and pre-planning skills, such as problem-solving) and knowledge (e.g., about available resources and opportunities and how to access them in their communities); and (d) develop strategies to address specific fears or barriers to participation and modify leisure pursuits (e.g., learning to use adaptive aids). Rehabilitation practitioners are ideally positioned to deliver leisure education, with example programs and content readily available (33–35).

## Conclusion

The intended outcome of many CCSM programs is to increase the abilities of clients/patients to self-manage their chronic conditions within the context of their daily activities and responsibilities, so that they are able to do the things they need or want to do in a way that supports their wellness. Integral to this, although often not explicitly stated, is to ensure people living with chronic conditions find ways to engage in daily activities that are meaningful to them. Enjoyable, personally meaningful leisure pursuits that support efforts to proactively manage one's condition and foster a sense of self-efficacy are viewed by people living with chronic conditions as particularly beneficial to their health and wellbeing (22, 24, 43, 44) and to self-managing their health condition. There is a risk that, without explicit attention to leisure education and subsequent leisure engagement, “meaningful” in this context narrowly focuses on activities that are primarily framed as important for immediate disease management without much consideration for the meaningful (i.e., valued) activities that provide the autonomy and agency of leisure (e.g., sense of mastery, belonging) (39). Because of its potential accessibility (e.g., can be done in one's home, neighborhood, or community), leisure is a particularly potent resource for CCSM, especially in rural and remote settings where diverse community-based leisure opportunities exist (10) but where access to formal rehabilitation services is typically more limited.

While the possibility of leisure engagement is implied in the TEDSS model, its potential to support self-management, especially in rural and remote communities, must be amplified. We believe that the TEDSS—and rehabilitation services for people living with chronic conditions in rural and remote communities—can be used to strengthen practices focused on leisure as a highly accessible resource for CCSM. As a starting point, we provided practical recommendations for strengthening rehabilitation services and CCSM supports to people living with chronic conditions in rural communities, including educating service providers and individuals, and building partnerships with a variety of community service providers. This approach requires challenging rehabilitation practitioners' views and values of leisure, developing supports in the community for leisure-based CCSM, and then addressing the knowledge and beliefs of individuals living with chronic conditions and their family/carers.

Inherent in supporting client/patient self-determination and patient-centered approaches to care is recognizing patients' motivations, needs, and strengths. It is therefore likely that aspects of most rehabilitation practitioners' current work encompass some leisure-related activities, whether this is helping re-establish or maintain valued activities and social or community connections. Therefore, inclusion of leisure likely exists, but is unnamed. Because it is unnamed, it is also likely an undervalued resource to accomplish clients/clients', family/carers' and health professionals' goals. We hope this *Perspective* piece serves as a call to action to rehabilitation and other health care practitioners to explicitly endorse a focus on leisure-based CCSM, and to intentionally work toward incorporating this complementary approach within their scope of practice. As immediate next steps, rehabilitation practitioners are encouraged to:

- Become better informed about the benefits of leisure for CCSM, and about community-based resources.
- Gather and begin to use tools for supporting leisure-based CCSM, including assessment tools/processes (e.g., TEDSS).
- Begin to build connections with community (informal and formal) recreation service providers.
- Explicitly engage clients/patients and family/carers in conversations and goal-setting about leisure and CCSM.

Leisure education tools and resources are integral to all of these steps.

There is growing recognition of the value of and need for leisure-related education and supports related to CSSM (36, 40) and this *Perspective* piece advances this area of work. Finally, while there is supporting evidence that incorporating leisure and leisure education into rehabilitation practice can enable healthier outcomes, specific evidence for supporting leisure-based CCSM in rural and remote settings is currently limited. Thus, there is a need to generate evidence to understand the impact and feasibility of coordinated, collaborative service provision that integrates leisure-based chronic condition self-management within existing models of care in rural and remote contexts.

## Data Availability Statement

The original contributions presented in the study are included in the article/supplementary material, further inquiries can be directed to the corresponding author.

## Author Contributions

All authors listed have made a substantial, direct, and intellectual contribution to the work and approved it for publication.

## Funding

Funding for this project was provided through an internal Research Development Grant from the Faculty of Health, Dalhousie University.

## Conflict of Interest

The authors declare that the research was conducted in the absence of any commercial or financial relationships that could be construed as a potential conflict of interest.

## Publisher's Note

All claims expressed in this article are solely those of the authors and do not necessarily represent those of their affiliated organizations, or those of the publisher, the editors and the reviewers. Any product that may be evaluated in this article, or claim that may be made by its manufacturer, is not guaranteed or endorsed by the publisher.
